# Mental Toughness and Associated Personality Characteristics of Marathon des Sables Athletes

**DOI:** 10.3389/fpsyg.2019.02259

**Published:** 2019-10-04

**Authors:** Keith Goddard, Claire-Marie Roberts, Liam Anderson, Lindsay Woodford, James Byron-Daniel

**Affiliations:** ^1^Zeus Ltd., Performance Psychology, Bradford-on-Avon, United Kingdom; ^2^Department of Psychology, University of the West of England, Bristol, United Kingdom

**Keywords:** ultra-endurance, NEO PI-R, big five factor personality, sport psychology, psychometrics

## Abstract

Mental toughness (MT) is commonly referred to as an important prerequisite for sustained athletic achievement. The increased research focus on MT has led to the development of a consistent debate centered around whether the construct is a unidimensional or multidimensional trait, and whether it can be differentiated from similar constructs such as hardiness. In order to move toward more clarity of MT, the present study is exploratory in nature, using athletes who have competed in the Marathon des Sables (MdS) ultra-endurance event. The MdS is a timed 250 km race in the Sahara Desert that takes place over 6 days in temperatures exceeding 40°C. Forty two British MdS competitors were recruited via the United Kingdom organizing company. Each participant completed the NEO PI-R as a measure of the five major domains of personality, as well as the six traits or facets that define each domain. Additionally, they completed the Sport Mental Toughness Questionnaire (SMTQ). The MdS sample’s NEO PI-R results were compared against general population norms, and results showed a distinct ultra-endurance athlete profile characterized by significantly higher levels of extraversion and openness to experience. Additionally, the MdS sample’s SMTQ scores were higher than the normed sample consisting of a collection of athletes representing multiple sports. Finally, linear regression analyses indicated a convergence between the two measures, supporting the argument that MT may in fact be measured by a general personality questionnaire such as the NEO PI-R.

## Introduction

The requirement to face adversity and overcome challenges is ever-present in modern society; from businesspeople achieving their performance targets to students under the extreme pressure of final exams (Gucciardi et al., 2015). One population to experience these adversities and challenges more than others are athletes (Gucciardi et al., 2017). High-performance sport is characterized by a demand to excel at optimal levels while performing under conditions that are considered exceptionally demanding (Jones et al., 2007). Psychological attributes such as self-confidence and the ability to cope with and interpret anxiety-related symptoms as positive are now commonly accepted as being major contributors to sporting success (Hanton and Fletcher, 2005). Nonetheless, mental toughness (MT) is commonly referred to as the “defining” attribute that enables one to thrive in these situations (Weinberg, 2010). Mental toughness has come to be widely viewed as an important prerequisite for sustained athletic achievement (Sheard, 2010) and it is consistently reported that higher levels of this construct in elite athletes is related to successful performance (Mahoney et al., 2014). However, research into successful sport performance has moved beyond just the elite, exploring both collegiate and youth athlete samples. Collectively, this range of participants indicates that MT is required across many sports and many achieving sport performers, not just elite athletes (Weinberg et al., 2017). It is said that mentally tough athletes are seen to experience less intense emotions and are viewed as being able to perform effectively in situations that are stressful (Crust, 2009) – an ideal trait to possess when considering the demanding lifestyle athletes experience (Hanin, 2010). However, it is likely that there are dysfunctional outcomes associated with high levels of mental toughness. Indeed, a study by Sabouri et al. (2016) found significant associations between the “dark triad” Sabouri et al. (2016, p. 229) of personality characteristics: Machiavellianism (a manipulative, self-centered, immoral, and calculative attitude), narcissism (exaggerated self-love, dominant, self-aggrandized, egocentric), and psychopathy (lack of empathy, adventurousness, low anxiety, cold-blooded), MT and vigorous physical activity. The authors suggest that a strong goal orientation and a high level of self-confidence may mean an individual is more likely to engage in vigorous physical activity or that MT and dark triad traits are a natural result of training for competitive sports.

The consensus that MT is a crucial factor for athletic excellence has increased interest toward not only developing a comprehensive understanding of the construct, but developing appropriate instruments to assess MT. Consequently, MT has become one of the most prevalent concepts within the broader field of positive psychology (Rusk and Waters, 2013). In fact, the academic focus on MT has risen at an exponential rate since the turn of millennia (Gucciardi et al., 2015). Several studies have been conducted investigating the definition of MT (Coulter et al., 2010) the process of building MT (Weinberg and Butt, 2011) and even potential theoretical explanations for MT (Harmison, 2011). However, this has, understandably been met with rigorous scrutiny. In fact, it could be argued that MT has become a quandary with ever-present issues and deliberations embedded into the literature. This includes a consistent debate over its categorization as a unidimensional or multidimensional trait (Coulter et al., 2016). This debate, though recently favoring the former option, has still not reached a consensus and this has not been helped by a lack of an agreed conceptualization of what MT is, and what it is not, with various outsourced definitions amongst the literature (Coulter et al., 2010; Clough and Strycharczyk, 2012). This has resulted in an endless list of positive psychological characteristics being associated with mental toughness which have unfortunately been justified via anecdotal evidence and personal accounts (Jones et al., 2007). This has even raised concerns amongst academics that the likelihood of defining MT in a concise and unambiguous way is diminishing (Bauman, 2016). Unfortunately, the issues surrounding MT are not purely restricted at the conceptual level.

Further apprehension has arisen surrounding the measurement of MT (Gucciardi et al., 2017). This concern centers around the relationship between MT and another similar construct of hardiness. The prevalent concern in this respect is that the commonly used measures of MT are faulty as they fail to diverge between MT and hardiness (Brand et al., 2014). This includes the MT48, the shorter MT18 and even the revised MTQ 48 (Crust, 2008). This leads to not only scrutiny of these measures but questions over the distinctiveness of both facets. Therefore, there is a need for greater clarification of what MT consists and does not consist of, its relationship with other personality traits and a valid measurement tool that emphasizes the distinctiveness of the facet.

On the topic of the need for a valid measurement tool, the Sport Mental Toughness Questionnaire (SMTQ; Sheard et al., 2009) appears to be a significant step forward in offering a psychometrically robust measure of general sport mental toughness and most importantly triumphs where the others falter demonstrating divergent validity from hardiness (Sheard, 2010). In relation to the need for greater clarification of what MT consists and does not consist of and its relationship with other personality traits, the Marathon des Sables (MdS) presents an opportune example for this exploration. The event is a timed 250 km race completed over 6 days across the Sahara Desert in temperatures well above 40°C (Knoth et al., 2012). In focusing on extreme ultra-endurance athletes, where a significant proportion of success is likely to be due to MT (Zeiger and Zeiger, 2018) due to the lack of extrinsic reward for many competitors, a clearer and more detailed insight may be gained of the characteristics that underpin what is termed MT. This is emphasized by several accounts in the press that frequently note that individuals finish the MdS regardless of position. Additionally, measuring detailed general personality alongside MT may give a clearer insight into the characteristics that are crucial for overcoming significant challenges in sporting endeavor, as well as adding some clarity to the nature of what is perhaps an overused and equivocal term – MT.

The present study is exploratory in nature and aims to build on and add to the current body of research exploring the characteristics that enable athletes to deal with the challenges they may face in sport. In doing so this research strives to achieve three aims. The primary aim is to describe the personality characteristics, including MT, of extreme ultra-endurance athletes. A secondary aim is to gain greater insights into MT and its independence as a separate personality trait. Vicariously, a third aim is to start establishing sport-specific norms on a measure of general personality (NEO PI-R) to allow holistic assessment and development of athletes as well as continuing to establish additional construct and divergent validity of a measure of general sport MT (SMTQ). The NEO PI-R provides a comprehensive and detailed assessment of adult personality based on the Five-Factor Model, which has been previously found to correlate with mental toughness constructs (Horsburgh et al., 2009; Delaney et al., 2015).

## Materials and Methods

### Participants and Design

Participants were 42 British athletes (33 Male, 9 Female) aged between 28 and 57 years (M = 42, SD = 8.15) who had successfully completed the MdS in either 2009 or 2010, achieving race positions between 78th and 891st. A convenience self-selecting sampling approach was used in which ethical approval was acquired prior to recruitment from the ethics committee of London Metropolitan University. Participants were recruited via an open invitation to take part in the study. This was sent to the United Kingdom organizing company of the MdS, who then forwarded the invitation to all competitors. A similar invitation was also posted on the United Kingdom MdS networking site. The term mental toughness was not mentioned in either invitations to avoid preconceived bias. The design of the present study consisted of cross-sectional group differences where the participants were compared to the test norm group of the NEO PI-R (general population) and the test norm group of the SMTQ (male and female).

### Materials

#### NEO PI-R

The NEO PI-R (McCrae and Costa, 1987) measures five factors of personality [Neuroticism (N), Extraversion (E), Openness to Experience (O), Agreeableness (A) and Conscientiousness (C)] with six traits underpinning each factor (see Table 1). The NEO-PIR consists of 240 items rated on a 5-point Likert scale rated from strongly agree to strongly disagree. Examples of items are as follows: 1 “I am not a worrier” and item 12 “I am dominant, forceful, and assertive.” The NEO PI-R is considered psychometrically robust with alpha coefficients generally over 0.70 and ranging between 0.58 and 0.92 (Costa and McCrae, 2006).

**TABLE 1:** The five factors of personality and their underpinning traits measured in the NEO-PIR.

**Factors**						
Neuroticism	Anxiety (N1)	Hostility (N2)	Depression (N3)	Self-consciousness (N4)	Impulsiveness (N5)	Vulnerability (N6)
Extraversion	Warmth (E1)	Gregariousness (E2)	Assertiveness (E3)	Activity (E4)	Excitement seeking (E5)	Positive emotion (E6)
Openness	Fantasy (O1)	Esthetics (O2)	Feelings (O3)	Actions (O4)	Ideas (O5)	Values (O6)
Agreeableness	Trust (A1)	Straightforwardness (A2)	Altruism (A3)	Compliance (A4)	Modesty (A5)	Tender-mindedness (A6)
Conscientiousness	Competence (C1)	Order (C2)	Dutifulness (C3)	Achievement-striving (C4)	Self-achieving (C5)	Deliberation (C6)

#### The Sports Mental Toughness Questionnaire

The SMTQ (Sheard et al., 2009) measures General Mental Toughness (G_MT_) and its sub-facets of Confidence (CF), Constancy (CS), and Control (CT). It consists of 14 items rated on a 4-point Likert Scale ranging from “not at all true” to “very true.” An example of an item is as follows: “I am committed to completing the tasks I have to do.” The measure has good internal consistency with alpha coefficients ranging from 0.7 to 0.8 (Sheard, 2010) and most importantly has been shown to have moderate divergent validity with hardiness, dispositional optimism and affect (Sheard, 2010).

### Procedure

A total of 65 participants expressed a desire to take part in the study and were sent an additional email containing a consent form, a personal details (demographic) form and links to the NEO PI-R and SMTQ with instructions for completion. Specifically, the participants were instructed not to consider the questions on either measure in great depth, and to respond to the questions honestly. They were given 4 weeks to complete the measures in which reminders were sent out prior to the deadline and non-returners were contacted twice after the closing date. 42 participants completed both the NEO PI-R and SMTQ with the nine who solely completed the former excluded from analysis. The NEO PI-R results were auto-downloaded via computer whereas the SMTQ results were emailed by return and hand scored.

### Data Analysis

Two independent sample *t*-tests were carried out to compare the means of two independent groups in order to determine whether there is statistical evidence that the associated population means are significantly different: the first using the mean scores of the NEO PI-R MdS group and the NEO PI-R test’s general population norms; the second using the mean scores of the SMTQ sample and compared to the mean scores of the test norms (male and female). Additionally, a Pearson correlation test was conducted on the NEO PI-R (five dimensions and 30 traits) and SMTQ (G_MT,_ CF, CT, and CS) scores to determine the strongest correlations present between the two constructs. Finally, linear regressions were conducted to examine variance in SMTQ as predicated by five factors and 30 traits of NEO PI-R.

## Results

### Descriptive Statistics and Normal Distribution

To test the normality of data, Komologorov–Smirnov (K–S), and tests of skew and kurtosis were carried out. Results indicated the NEO PI-R traits of E6 (positive emotion), O3 (feelings) and C2 (Order) (*p* < 2.58), and E3 (assertiveness) (*z* score = 2.88, *p* < 3.29) as well as C5 (self-achieving) were skewed (*p* < 2.58). Therefore, caution would be exercised in interpreting the data associated with these constructs. Means and standard deviations SMTQ variables are presented in Table 2 and NEO PI-R variables are presented in Table 3.

**TABLE 2:** Means and standard deviations of SMTQ.

	**Gender**				**Total**	
	**Male**		**Female**			
	***M***	***SD***	***M***	***SD***	***M***	***SD***
Confidence	18.21	2.29	16.22	1.86	17.79	2.33
Control	11.70	2.46	10.56	2.74	11.45	2.54
Constancy	14.03	1.47	14.67	1.32	14.17	1.45
G_MT_ total	43.94	4.71	41.44	3.94	43.40	4.63

**TABLE 3:** Means and standard deviations of NEO PI-R questionnaire.

**Factor/Trait**		

	***M***	***SD***
N	77.07	19.50
E	122.93	16.98
O	125.07	16.75
A	119.48	15.84
C	122.62	17.36
N1	12.40	5.06
N2	12.67	4.43
N3	12.95	5.14
N4	13.48	4.36
N5	17.24	4.60
N6	8.33	3.98
E1	22.95	3.95
E2	16.17	5.29
E3	19.07	3.97
E4	21.90	3.82
E5	20.10	4.49
E6	22.74	4.54
**Trait**		
O1	20.02	4.29
O2	17.79	5.50
O3	23.05	4.61
O4	19.90	3.08
O5	21.31	5.52
O6	23.00	2.54
A1	21.45	3.94
A2	18.52	4.19
A3	24.19	3.47
A4	16.60	4.14
A5	18.43	4.53
A6	20.29	2.88
C1	22.71	3.29
C2	17.55	4.51
C3	23.4	2.86
C4	21.52	4.34
C5	21.19	4.42
C6	16.24	3.68

### Marathon des Sables Group Compared to Test Population Norm Groups

A series of independent sample *t*-tests were conducted on the mean scores of the NEO PI-R MdS group sample and the mean scores of the NEO PI-R general population norms. Significant differences were observed in two of the five factor personality traits – extraversion (*t*_(__41__)_ = 5.16, *p* = 0.00) and openness to experience (*t*_(__41__)_ = 5.60, *p* = 0.00) and in 17 out of 30 of the underpinning traits. The significant results are presented in Table 3. In summary, MdS athletes reported significantly lower levels of anxiety (N1), vulnerability (N6), straightforwardness (A2), order (C2), and deliberation (C6). Conversely, the MdS athletes reported significantly higher impulsiveness (N5), assertiveness (E3), activity (E4), excitement seeking (E5), positive emotions (E6), fantasy (O1), feelings (O3), actions (O4), ideas (O5), values (O6) than the general population norms (Table 4).

**TABLE 4:** NEO PI-R factors and trait comparisons of the MdS group with general population norm group.

**Factor/Trait**					**99% CI of the difference**	
	
	***t***	**df**	**Sig. (2-tailed)**	**Mean difference**	**Lower**	**Upper**
E	5.164	41	0.000^∗^	13.529	6.45	20.61
O	5.598	41	0.000^∗^	14.471	7.49	21.45
N1	–2.427	41	0.020^∗^	–1.895	–4.00	0.21
N5	2.028	41	0.049^∗^	1.438	–0.48	3.35
N6	–2.714	41	0.010^∗^	–1.667	–3.33	–0.01
E3	5.346	41	0.000^∗^	3.271	1.62	4.92
E4	7.307	41	0.000^∗^	4.305	2.71	5.9
E5	5.337	41	0.000^∗^	3.695	1.82	5.57
E6	3.621	41	0.001^∗^	2.538	0.64	4.43
O1	5.178	41	0.000^∗^	3.424	1.64	5.21
O3	3.866	41	0.000^∗^	2.748	0.83	4.67
O4	7.368	41	0.000^∗^	3.505	2.22	4.79
O5	2.712	41	0.010^∗^	2.310	0.01	4.61
O6	5.874	41	0.000^∗^	2.300	1.24	3.36
A2	–4.143	41	0.000^∗^	–2.676	–4.42	–0.93
A4	–3.609	41	0.001^∗^	0.41	–2.305	–0.58
C2	–2.086	41	0.043^∗^	–1.452	–3.33	0.43
C4	3.011	41	0.004^∗^	2.017	0.21	3.83
C6	–9.792	41	0.000^∗^	–5.562	–7.10	–4.03

*^∗^*p* < 0.05.*

A series of independent sample *t*-tests were conducted on the mean scores of the SMTQ sample and compared to the means scores of the tests’ norms (male and female). There were significant differences noted across the male and female MdS population in comparison with the normed data in general mental toughness (G_MT_), confidence (CF), and Constancy (CS). See Table 5 for the full results.

**TABLE 5:** Comparisons of the MdS group males and females SMTQ scores with SMTQ test norm group.

**SMTQ scale**					**99% CI of the difference**	
	
	***t***	**df**	**Sig. (2-tailed)**	**Mean difference**	**Lower**	**Upper**
**Males**						
G_MT_	3.731	32	0.001^∗^	3.059	0.81	5.30
CF	2.968	32	0.006^∗^	1.182	0.09	2.27
CS	4.345	32	0.000^∗^	1.110	0.41	1.81
**Females**						
G_MT_	2.432	8	0.041^∗^	3.194	−1.21	7.60
CF	2.541	8	0.035^∗^	1.572	−0.50	3.65
CS	3.802	8	0.005^∗^	1.677	0.20	3.16

*^∗^*p* < 0.05.*

### Linear Relationship of NEO-PI-R and SMTQ

A series of Pearson correlations between NEO PI-R (five traits and 30 underpinning traits) and SMTQ (total mental toughness and three sub-scales) indicated a number of significant relationships between constructs. Pearson correlations revealed moderate to strong relationships between a number of NEO PI-R and SMTQ scores for the MdS group (Table 6).

**TABLE 6:** Pearson intercorrelations between SMTQ and the factors and traits of NEO PI-R.

**NEO PI-R factor**	**SMTQ**			
	**Confidence**	**Control**	**Constancy**	**GMT tot**
Neuroticism	–0.497^∗∗∗^	–0.578^∗∗∗^	–0.396^∗∗^	–0.692^∗∗∗^
Extraversion	0.348^∗^	–	–	–
Openness	0.456^∗∗^	0.338^∗^	–	0.503^∗∗∗^
Agreeableness	–	–	–	–
Conscientiousness	0.512^∗∗∗^	0.336^∗^	0.539^∗∗∗^	0.611^∗∗∗^
**NEO PI-R Trait**				
N1	–0.461^∗∗^	–0.504^∗∗∗^	−0.363^∗^	–0.623^∗∗∗^
N2	–	–0.583^∗∗∗^	–	–0.470^∗∗^
N3	–	–0.477^∗∗∗^	–0.438^∗∗^	–0.520^∗∗∗^
N4	–0.498^∗∗∗^	–0.437^∗∗^	–	–0.541^∗∗∗^
N5	–	–	–	–
N6	–0.615^∗∗∗^	–0.438^∗∗^	–0.438^∗∗^	–0.687^∗∗∗^
E1	–	–	–	–
E2	–	–	–	–
E3	0.421^∗∗^	–	–	–
E4	0.436^∗∗^	–	–	–
E5	–	–	–	–
E6	–	–	–	0.320^∗^
O1	–	–	–	–
O2	0.316^∗^	–	0.345^∗^	0.318^∗^
O3	0.389^∗^	–	–	–
O4	–	–	–	–
O5	–	0.387^∗^	–	0.453^∗∗^
O6	0.622^∗∗∗^	0.435^∗∗^	0.385^∗^	0.673^∗∗∗^
**NEO PI-R Trait**				
A1				
A2				
A3				
A4	−0.365^∗^			
A5				
A6				
C1	0.590^∗∗∗^	0.478^∗∗∗^	0.539^∗∗∗^	0.728^∗∗∗^
C2				
C3	0.431^∗∗^	0.307^∗^	0.320^∗^	0.486^∗∗∗^
C4	0.486^∗∗∗^		0.408^∗∗^	0.526^∗∗∗^
C5	0.413^∗∗^		0.502^∗∗∗^	0.502^∗∗∗^
C6			0.496^∗∗∗^	0.421^∗∗^

*All non-significant (*p* > 0.05) correlations removed. ^∗^*p* < 0.05, ^∗∗^*p* < 0.01, ^∗∗∗^*p* < 0.001.*

A series of linear multiple regressions was undertaken to examine variance in SMTQ as predicated by five factors and 30 traits of NEO PI-R-utilizing the six strongest correlations for each factor of SMTQ. Predictors were loaded into the model using forward stepwise method, outlier removal was set at casewise 2SD to reduce potential type I errors. For G_MT,_ Neuroticism, Openness, and Conscientiousness predicted 58.2% of the variance (Adj. *R*^2^) and at underpinning trait level, C1 (Competence), O6 (Values), and N6 (Vulnerability) accounted for 66.5% of the overall variance. For Confidence (CF), Conscientiousness, and Openness explained 38.6% of the overall variance whereas at trait level, N6 (Vulnerability), O6 (Values), and A4 (Compliance) explained 56.1% of the overall variance. For Control (CT), Neuroticism explained 31.8% of the overall variance and at trait level, N2 (Hostility) and N1 (Anxiety) accounted for 41.4% of the overall variance. For Constancy (CS), Conscientiousness explained 27.2% of the overall variance and at trait level, C1 (Competence) and C6 (Deliberation) explained 35.0% of the overall variance.

## Discussion

This study set out to achieve three aims: (1) to describe the personality characteristics of extreme ultra-endurance athletes, (2) gain a greater insight into MT and its independence as a personality trait, and (3) to establish sport-specific norms on a measure of general personality (NEO PI-R) and general sport MT (SMTQ). With this in mind, the results indicated that ultra-endurance athletes appeared to have distinct profile of personality traits compared to the general population. Additionally, their mental toughness profile was differentiated from fellow athletes. Specifically, on the big five factor personality traits, MdS athletes demonstrated a statistically significantly higher level of extraversion and openness to experience. This finding adds to the contradictory data on the differences in personality traits between athletes and non-athletes (Malinauskas et al., 2014). The equivocal findings identifying potential differences in extraversion between these two populations is supported by studies that have found no differences (e.g., Vealey, 2002; McKelvie et al., 2003), with others reporting a consistently higher level of extraversion than non-athletes (e.g., Egloff and Gruhn, 1996), albeit with a small effect size (Cohen’s *d* = 0.33). Having said that, a more recent review suggests that athletes are characterized by higher levels of extraversion compared with non-athletes (Hughes et al., 2003). This is an important observation given that extraversion is associated with low stress reactivity (Connor-Smith and Flachsbart, 2007), coping (Nicholls and Polman, 2007), and mental toughness (Egan and Stelmack, 2003). The second big five personality factor that differentiated between MdS athletes and the general population was openness to experience. To recap, openness to experience reflects an individual’s ability to seek out new experience and a general preference for an active imagination (fantasy), esthetic sensitivity, attentiveness to inner feelings, preference for variety, and intellectual curiosity (Duriez et al., 2004; Allen et al., 2013). The presence of a higher level of openness may play a greater role in predicting participation in non-traditional sports due to the level of receptiveness to ideas and opportunities for new experiences (Wilson and Dishman, 2015). Additionally, this finding may reflect a higher propensity for ultra-endurance runners tend to focus on the wonder of the external environment, driven by the preference for esthetic sensitivity (Acevedo et al., 1992; Baker et al., 2005). However, the findings regarding the differences on the openness trait differ from a recent study examining the personality profiles of athletes who have experienced most success in their career (Steca et al., 2018). This study concluded that the most successful athletes showed consistently higher scores across all big-five personality dimensions apart from Openness to Experience.

In making sense of the MT results the MdS athletes’ scores on the SMTQ suggest that this population are likely to be more confident than other athletes yet surprisingly MdS athletes’ confidence (CF) levels are comparable to that of the general population. This is particularly intriguing as self-confidence is commonly cited (e.g., Bull et al., 2005; Golby et al., 2007; Crust, 2008; Gucciardi and Gordon, 2009) as being the primary theme for mental toughness and therefore it would appear, based on this theoretical approach, that the MdS sample possessed no more mental toughness than their non-sporting counterparts despite the demands of their event. Remarkably, it was additionally noted that the SMTQ was not related to extraversion (E) and yet it was a characteristic that differentiated the MdS group from the general population. However, this is not the only result from the current study to question previous findings. For example, Horsburgh et al. (2009) stated that mentally tough individuals are more likely to be outgoing and sociable. In fact, social support is seen as key in developing mental toughness (Connaughton and Hanton, 2009). However, these conclusions were not supported by the findings of the current study. This could be an indication of the true uniqueness of the current sample with ultra-endurance events attracting only solitary people, but it is also a signal of the importance of utilizing in-depth personality measures as well as sport-specific norms on general measures in future research.

Another Big 5 trait to have a strong link to mental toughness is neuroticism (N) which also has relationships with both coping and hardiness (Cerin, 2004; Maddi, 2006) yet intriguingly, in the present study, there were no significant differences between the MdS sample and the general population on this higher order construct. However, the MdS athletes reported significantly lower levels of anxiety and vulnerability. However, this raises the question whether feeling free-flowing anxiety and experiencing frustration and anger states (if present) are facilitative or debilitative to performance based on the interpretation at that time. A key question for this population could be “*Do MdS athletes find ultra-endurance events stressful?*.” This highlights the possibility that the demands athletes have to cope with may be so distinct that they require different dispositions to deal with them.

In regard to the second and third aim of the present study, the results provide insight into the nuances of mental toughness as well as its potential as a sole personality trait. For example, the results indicated a number of moderate to strong relationships between the SMTQ, its subscales and three of the factors and seven of the traits of NEO P-IR. The three NEO P-IR factors (N, O, C) accounted for 58.2% of the variance of SMTQ Total Mental Toughness (G_MT_) whilst three out of the thirty NEO P-IR traits (N6, O6, C1) accounted for 66.5% of the variance of Mental Toughness (G_MT_). This not only indicates a level of convergence between the two measures but it may also provide an argument to counter the views that mental toughness cannot be measured through a general personality measure, and that it is a separate trait (Bull et al., 2005; Gucciardi and Gordon, 2009). In terms of its relationship with the NEO PI-R, the assumed conceptual link would be between Constancy (CS) and Conscientiousness (C) given their definitions. This was supported by the results; however, closer scrutiny suggests that only two underpinning traits within Conscientiousness load onto constancy; competence (C1) and deliberation (C6). It is peculiar given the conceptualization of constancy, why it is not more strongly related to conscientiousness especially as another logically assumed trait in achievement striving (C4) did not significantly relate to constancy either. This was not the only surprising result to emerge from the SMTQ and NEO PI-R analysis. For example, Vulnerability (N6) accounted for a significant amount of variance in SMTQ mental toughness (G_MT_) as well as confidence (CF) but surprisingly, not in control (CT). This suggests that confidence may be measuring aspects of control and control itself, is not wholly measuring what it purports to measure. In other words, its construct validity is brought into question. It is not transparent from the original SMTQ research whether control is intended to measure both perceived ability to cope and the ability to control emotions, despite both being cited within the research (Sheard et al., 2009; Sheard, 2010). Given its relationship with anxiety (N1) and angry hostility (N2) on NEO PI-R and lack of relationship with vulnerability (N6) it would appear to be the latter only. This confusion is thrown into further disrepute considering the initial item bank for the SMTQ was generated using an extremely limited sample utilizing small number of athletes from a small range of sports and therefore could explain the issues of validity. Gaining clarity over this is important especially if the SMTQ is to become influential concerning the strategies and interventions used to develop athletes.

### Strengths and Limitations

There are strengths inherent within the study and some limitations that need to be considered for future research. Technically the use of a dual instrument approach (personality and mental toughness) was seen as important for the empirical rigor of investigating mental toughness. In addition, the sample choice of a mixed ability group moves mental toughness research away from being dominated by elite only studies, something that future research needs to continue to replicate. In terms of personality measures, the study has begun to build sport-specific norm bases for the NEO PI-R as well as continuing the ongoing validation of the SMTQ within a specific sport. However, it must be considered that the small, British and male dominated sample in the current study led to a restricted choice of statistical analyses and increased the need to adopt a cautious approach to it in order to minimize Type I errors, which may have increased the possibility of Type II errors. Additionally, as the study was conducted 3 or 15 months after the event, no baseline measures were taken prior to the event for comparative purposes and further analysis. Cross-comparisons between the two tests may have been less robust, as different populations (the tests’ norm groups) were used as comparator groups, than if the same comparison group was used for both tests. Further limitations arise due to sole reliance on self-report questionnaires with no alternative forms of assessment or evaluations by third parties. This is particularly relevant to the NEO PI-R as its items are not sport-specific and therefore may not be suitable for athletes or in this case MdS populations. However, future research can aid in this as establishing separate norm bases on the NEO P-IR for specific sports will add to its utility and resultingly lead to a general sports norm base. This will be advantageous to the study and development of the characteristics that allow athletes to push themselves to extremes, including mental toughness.

### Future Directions

An examination of the results of the present study allows for the suggestion of future directions of related research. For example, future investigation should involve a comparison of athletes from different competitive levels as literature has indicated an over-reliance on elite athletes to inform mental toughness research (Golby et al., 2007; Crust, 2008; Sheard et al., 2009). Additionally by encouraging the development of sport-specific norms, general personality measures could provide a broader and more holistic understanding of athletes as well as developing a greater awareness of athletes, which is especially important in team contexts (Beauchamp et al., 2007); In fact NEO PI-R is seen to impact on other domains like orientation to people, thinking style, operational style and emotional style (Costa and McCrae, 2006; Rust and Lord, 2006; Lord, 2007). This will not only allow greater pragmatism when working with athletes to develop the characteristics that are required for them to become successful in training and competitions but relating specifically to the current research, it will be advantageous to the study and development of the characteristics that allow athletes to push themselves to extremes. Additionally, further investigation of participants’ openness scores could be addressed in future research to give an insight into the differences between those who use associative or dissociative strategies during ultra-endurance events, as this is an area of contention between authors (Acevedo et al., 1992; Baker et al., 2005).

## Conclusion

To conclude, the MdS has provided a unique opportunity to consider the personality characteristics of ultra-endurance athletes using both the NEO PI-R and the SMTQ. The NEO PI-R appears to add insight into the MdS population’s personality and mental toughness that perhaps a factor-only personality measure and the SMTQ alone could not achieve. Additionally, whilst this study has potentially added some validity to the SMTQ it has also raised some conceptual questions regarding the measure and the concept of mental toughness in general. This study has potentially supported the increasing call for sport psychology to reengage with personality research to understand how individuals deal and develop the ability to deal with the challenges they face across situations, especially competition, training and lifestyle demands (Gucciardi et al., 2008). The in-depth profile that results from completing the NEO PI-R is likely to be valuable to sport psychologists and practitioners in achieving this aim although this must coincide with the establishing of sport-specific norms through future research that acknowledges the huge variance that exists in the sporting population.

## Data Availability Statement

The datasets generated for this study are available on request to the corresponding author.

## Ethics Statement

The studies involving human participants were reviewed and approved by London Metropolitan University. The patients/participants provided their written informed consent to participate in this study.

## Author Contributions

KG designed and collected the data for the study and drafted the original manuscript. C-MR co-ordinated the preparation of the draft into a final version and checked and verified the statistical analysis. LA researched the most up to date literature on mental toughness, and added this to the manuscript. LW and JB-D acted as advisors and critical friends throughout the process.

## Conflict of Interest

KG owns and manages Performance Psychology, Zeus Ltd. The remaining authors declare that the research was conducted in the absence of any commercial or financial relationships that could be construed as a potential conflict of interest.
